# Coronal plane alignment of the knee classification system does not reliably predict tibiofemoral joint gaps in arthritic knees undergoing total knee arthroplasty

**DOI:** 10.1002/ksa.12734

**Published:** 2025-06-29

**Authors:** Ishaan Jagota, Rami M. A. Al‐Dirini, Mark Taylor, Joshua Twiggs, Brad Miles, David Liu

**Affiliations:** ^1^ 360 Med Care Sydney Australia; ^2^ Enovis ANZ Sydney Australia; ^3^ College of Science and Engineering Flinders University Adelaide Australia; ^4^ The Gold Coast Centre for Bone and Joint Surgery Palm Beach Queensland Australia

**Keywords:** CPAK, joint balance, joint gaps, preoperative planning, total knee arthroplasty

## Abstract

**Purpose:**

Achieving optimal mediolateral balance in extension and flexion is critical for improving outcomes in total knee arthroplasty (TKA). Knee classification tools, like coronal plane alignment of the knee (CPAK), do not directly consider the soft tissue profile of the joint. This study evaluated the variation in tibiofemoral gap measurements across CPAK phenotypes and examined the relationships between preoperative tibiofemoral joint gaps and the arithmetic hip‐knee‐ankle angle (aHKA) and joint line obliquity (JLO), important for TKA planning and execution.

**Methods:**

A retrospective analysis of 433 knees from the Joint Dynamics Registry computed tomography (CT) database was performed. Patients received preoperative long‐leg CT scans and extension and flexion distracted radiographs. The CT scans were segmented and landmarked to produce three‐dimensional bone models and derive anatomical measurements, including aHKA and JLO. The models were registered to the two distracted radiographs, and an osteophyte correction algorithm was applied to calculate the medial and lateral joint gaps in extension and flexion. Composite gap measurements (mean and difference) were also determined. Pearson's correlation and multivariate regression assessed the relationships between joint gaps and aHKA and JLO. ANOVA compared joint gaps across CPAK groups.

**Results:**

Small but statistically significant differences in joint gap measurements were observed between CPAK groups I and III, and II and III. Weak univariate correlations were observed between aHKA and joint gaps (*r* ≤ |0.32 |), with fewer statistically significant relationships for JLO (*r* ≤ |0.15 |). Multivariate regression explained only 10.2% and 1.4% of aHKA and JLO variance, respectively.

**Conclusion:**

While useful for describing coronal alignment, CPAK displayed limited predictive capability for preoperative tibiofemoral joint gaps in TKA patients. Direct assessment of joint gaps remains crucial for surgical planning. Future research should focus on integrating joint gap measurements with bony morphology in preoperative planning workflows to improve TKA personalisation.

**Level of Evidence:**

Level II.

**Abbreviations**
3D: three‐dimensional
aHKA: arithmetic hip‐knee‐ankle
CPAK: coronal plane alignment of the knee
CT: computed tomography
HKA: hip‐knee‐ankle
IQR: interquartile range
JLO: joint line obliquity
LDFA: lateral distal femoral angle
MPTA: medial proximal tibial angle
TKA: total knee arthroplasty

## INTRODUCTION

Recent total knee arthroplasty (TKA) alignment philosophies have shifted towards personalised approaches [18], aiming for better joint balance while minimising soft tissue release [1, 2]. Key to this balance is an understanding of the knee's soft tissue characteristics through tibiofemoral joint gaps and/or knee laxity and its relationship to alignment and bony morphology, which play a critical role in postoperative TKA stability [25, 33]. However, determining the optimal alignment strategy remains challenging due to high inter‐patient variability in both bony anatomy [11, 16, 20, 30] and soft tissue behaviour [5, 8, 10].

Knee classification systems are integral in characterising anatomical variations among TKA patients, providing a framework for alignment strategies. Traditional systems broadly classify coronal alignment as neutral, varus or valgus, often overlooking joint line obliquity (JLO) and may be influenced by assessment position or potential soft tissue degeneration [18]. The coronal plane alignment of the knee (CPAK) classification system addresses these limitations by categorising the knees using both the arithmetic hip‐knee‐ankle (aHKA) angle and JLO to create a more nuanced understanding of coronal knee alignment [19]. Although CPAK does not fully capture the three‐dimensional (3D) complexity of knee morphology [16] and phenotype distributions vary across gender and ethnicity [9, 14, 21, 22, 28], it remains widely used to guide alignment strategy and joint balance optimisation [7, 19]. Proponents assert that restoring a patient's CPAK category, aHKA and JLO not only re‐establishes constitutional alignment but results in balanced TKA gaps without the need for any adjustments or soft tissue release.

Despite its clinical utility, limited research has examined whether CPAK's phenotypes or coronal alignment parameters offer insight into the knee's soft tissue behaviour. This raises questions about whether CPAK alone offers a comprehensive framework for TKA planning without consideration to soft tissue factors. This is important to explore because preoperatively assessed joint laxity has been shown to predict postoperative outcomes [15] and such joint gap and laxity data can enable patient‐specific TKA planning to balance medial and lateral tibiofemoral gaps in extension and flexion [17]. Moreover, achieving joint gap and laxity balance targets can enhance postoperative patient outcomes [32, 33].

The primary aim of this study was to evaluate how preoperative medial, lateral and composite joint gap measurements in extension and 90° flexion vary across CPAK groups in a patient cohort undergoing primary TKA for osteoarthritis. The secondary aim was to assess the relationship between the joint gap measurements and aHKA and JLO. The study hypothesis was that statistically significant differences in joint gap measurements will be present between CPAK phenotypes and that the relationships between the joint gap measurements and aHKA and JLO are linear.

## METHODS

A total of 433 knees from 407 patients undergoing TKA between 29 April 2021 and 6 October 2023 were retrospectively analysed. Procedures were performed by 1 of 10 orthopaedic surgeons across 12 different orthopaedic centres. Patients were included if they were in the Joint Dynamics Registry computed tomography (CT) database and had undertaken preoperative extension distracted and flexion distracted radiographs. Joints with prior trauma or ligament injuries and revision or complex primary surgeries requiring revision or constrained prostheses were excluded. Patient demographics and preoperative coronal alignment parameters are outlined in Table 1.

**Table 1:** Patient demographics for the full study cohort and for the male and female subpopulations.

Measurement	All	Male	Female
Total joints	433	202	231
Left/right	210/223	98/104	112/119
Age (years)	70.3 (IQR 13.0)	69.7 (IQR 11.9)	70.8 (IQR 13.2)
CT HKA (° Varus)	4.0 (IQR 6.7)	5.3 (IQR 5.5)	3.4 (IQR 6.7)
MPTA (°)	86.4 (IQR 3.3)	86.1 (IQR 3.7)	86.7 (IQR 3.0)
LDFA (°)	86.7 (IQR 3.2)	87.0 (IQR 2.7)	86.4 (IQR 3.3)
aHKA (°)	−0.3 (IQR 4.6)	−0.9 (IQR 4.4)	0.3 (IQR 4.4)
JLO (°)	173.1 (IQR 4.4)	173.1 (IQR 4.4)	173.0 (IQR 4.5)

*Note*: Demographics are reported as amount or mean (interquartile range).

Abbreviations: aHKA, arithmetic hip‐knee‐ankle angle; CT, computed‐tomography; HKA, hip‐knee‐ankle angle; IQR, inter‐quartile range; JLO, joint line obliquity; LDFA, lateral distal femoral angle; MPTA, medial proximal tibial angle.

### Radiology capture and image processing

Preoperatively, each patient received a bilateral long‐leg CT scan and two distracted antero‐posterior radiographs: one each in extension and 90° flexion. The maximum CT slice thickness was 1.25 mm in the transverse plane and 1 mm in the coronal and sagittal planes. For the two radiographs, the knee was axially distracted using 2.5 kg ankle weights as described by Jagota et al. [17]. Trained engineers used Simpleware ScanIP (version M‐2017.06, Synopsys, Inc., Mountain View, USA) to segment and landmark the CT scans to create 3D bone models, which were then registered to each of the two radiographs. The segmentations, landmarks and registrations were all quality checked by a senior engineer to ensure accuracy. Such 3D model landmarking and measurement methods have demonstrated high reliability, with low variability between and within observers [6, 34].

### CPAK measurements

The bone models and landmarks were used to determine key coronal plane measurements required to calculate CPAK types. This included the medial proximal tibial angle (MPTA), defined as the medial proximal angle between the mechanical axis of the tibia and the line connecting the medial and lateral tibial wells, and the lateral distal femoral angle (LDFA), defined as the lateral angle between the mechanical axis of the femur and the line connecting the medial and lateral distal femoral condyles. These angles were used to calculate the aHKA (MPTA − LDFA) and JLO (MPTA + LDFA). As established by MacDessi et al., the aHKA was used to categorise the knee as neutral, varus or valgus and the JLO was used to categorise the knee as apex distal, neutral or proximal [19]. Together, the aHKA and JLO groupings defined the CPAK phenotype of each knee.

### Joint gap calculations and osteophyte correction

The native medial and lateral intercompartmental joint gaps were determined for each knee in the extension distracted and flexion distracted positions. These gaps were defined as the supero‐inferior distance between the most inferior point on the respective femoral condyle and a single plane across the proximal tibial plateau. The proximal tibial plane was defined using four reference landmarks: the medial and lateral edge landmarks of the proximal tibial surface, and the most anterior and posterior landmarks along the lateral proximal tibial plateau. The lateral compartment was selected for the identification of the anterior and posterior landmarks due to being less affected in the greater proportion of osteoarthritic joints [31]. These joint gap measurements were determined automatically using a custom script in RStudio (Posit, USA).

Ligament tenting due to the presence of osteophytes can result in the tightening of tibiofemoral joint gaps. Intraoperatively, such osteophytes are usually removed before gap or laxity assessment of the knee. To account for this, an osteophyte correction algorithm was applied as outlined in Jagota et al. [17]. The algorithm measured the difference between direct (straight‐line) and wrapped (shortest bony wrapped) ligament paths for the medial and lateral collateral ligaments. The difference in path lengths quantified the increase in medial and lateral joint gaps to account for expected osteophyte removal, which were added to the native gaps to determine the final medial and lateral joint gaps in extension and flexion. These four intercompartmental joint gap measurements were also used to determine the average extension gap, average flexion gap, extension gap difference and flexion gap difference for each patient, as defined in Table 2.

**Table 2:** Definitions for the four composite gap measurements.

Measurement	Definition
Average extension gap	$\frac{\left(\mathrm{Medial}\;\mathrm{extension}\;\mathrm{gap}\;+\;\mathrm{Lateral}\;\mathrm{extension}\;\mathrm{gap}\right)}{2}$
Average flexion gap	$\frac{\left(\mathrm{Medial}\;\mathrm{flexion}\;\mathrm{gap}\;+\;\mathrm{Lateral}\;\mathrm{flexion}\;\mathrm{gap}\right)}{2}$
Extension gap difference	Lateralextensiongap−Medialextensiongap Positive number denotes a larger lateral gap than medial gap
Flexion gap difference	Lateralflexiongap−Medialflexiongap Positive number denotes a larger lateral gap than medial gap

### Ethics approval

Ethics was approved by Bellberry Human Research Ethics Committee (Sydney, Australia, application number 2012‐03‐710).

### Statistical analysis

Descriptive statistics were employed to summarise the joint gap measurements for the full study cohort and for each CPAK phenotype. Data normality was evaluated subjectively using histograms and Q‐Q plots, and objectively by analysing skewness and kurtosis, and was determined to follow a normal distribution. The linear relationship between each of the eight joint gap measurements and aHKA and JLO were analysed using Pearson's correlation coefficients, with *p* < 0.05 indicating statistical significance. Separate multivariate linear regression analyses were performed to evaluate the ability of the four intercompartmental joint gaps (medial and lateral gaps in extension and flexion) to predict aHKA and JLO. Correlation coefficients of <0.4, 0.4–0.7 and >0.7 were considered weak, moderate and strong, respectively, and the strength of the correlation was used in the interpretation of clinical significance. One‐way analysis of variance was conducted to compare the mean values of the four joint gap measurements across the CPAK phenotypes. Where statistical significance (*p* < 0.05) was observed, post hoc Tukey's Honest Significance Difference tests were used to identify which pair(s) of phenotypes displayed statistically significantly different means for the given measurement.

## RESULTS

### CPAK distribution

**Figure 1:**
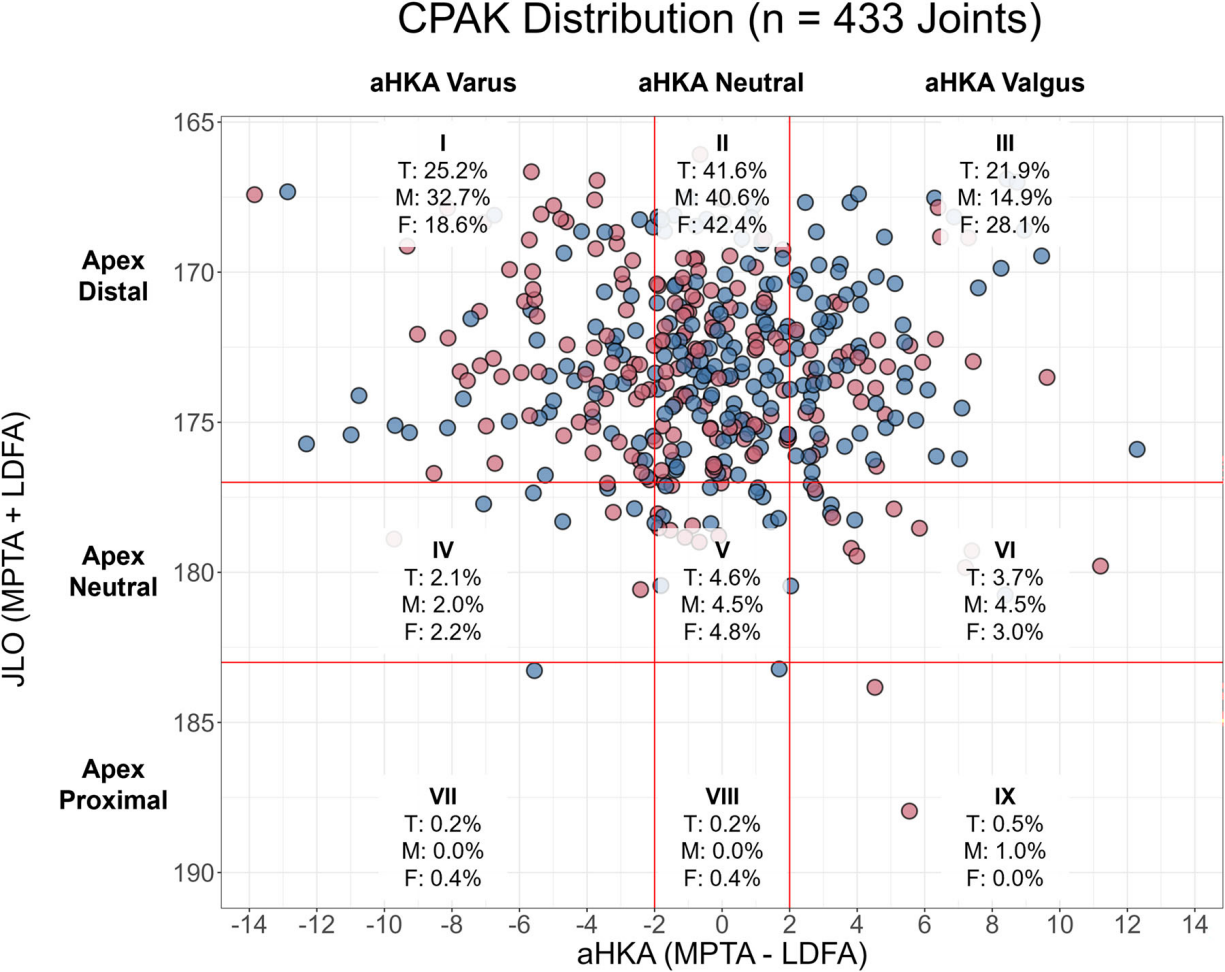
Preoperative CPAK distribution for the cohort of 433 knees, categorised by men (red) and women (blue). The proportion of knees categorised within each of the nine phenotypes is displayed for the overall cohort (T) as well as for the men (M) and women (F) subpopulations. aHKA, arithmetic hip‐knee‐ankle angle; CPAK, coronal plane alignment of the knee; JLO, joint line obliquity; LDFA, lateral distal femoral angle; MPTA, medial proximal tibial angle.

The CPAK distribution of the study patient population is displayed in Figure 1. As in previous studies, CPAK types VII, VIII and IX represented less than 1% of the study population and so were excluded from all statistical analyses [3, 13, 16]. CPAK type II (apex distal JLO and neutral aHKA) was most frequent across the study cohort (41.6%) as well as the men (40.6%) and women (42.4%) subpopulations. CPAK types I (25.2%) and III (21.9%) were the next most common across the study cohort.

### Joint gaps across CPAK Phenotypes

**Figure 2:**
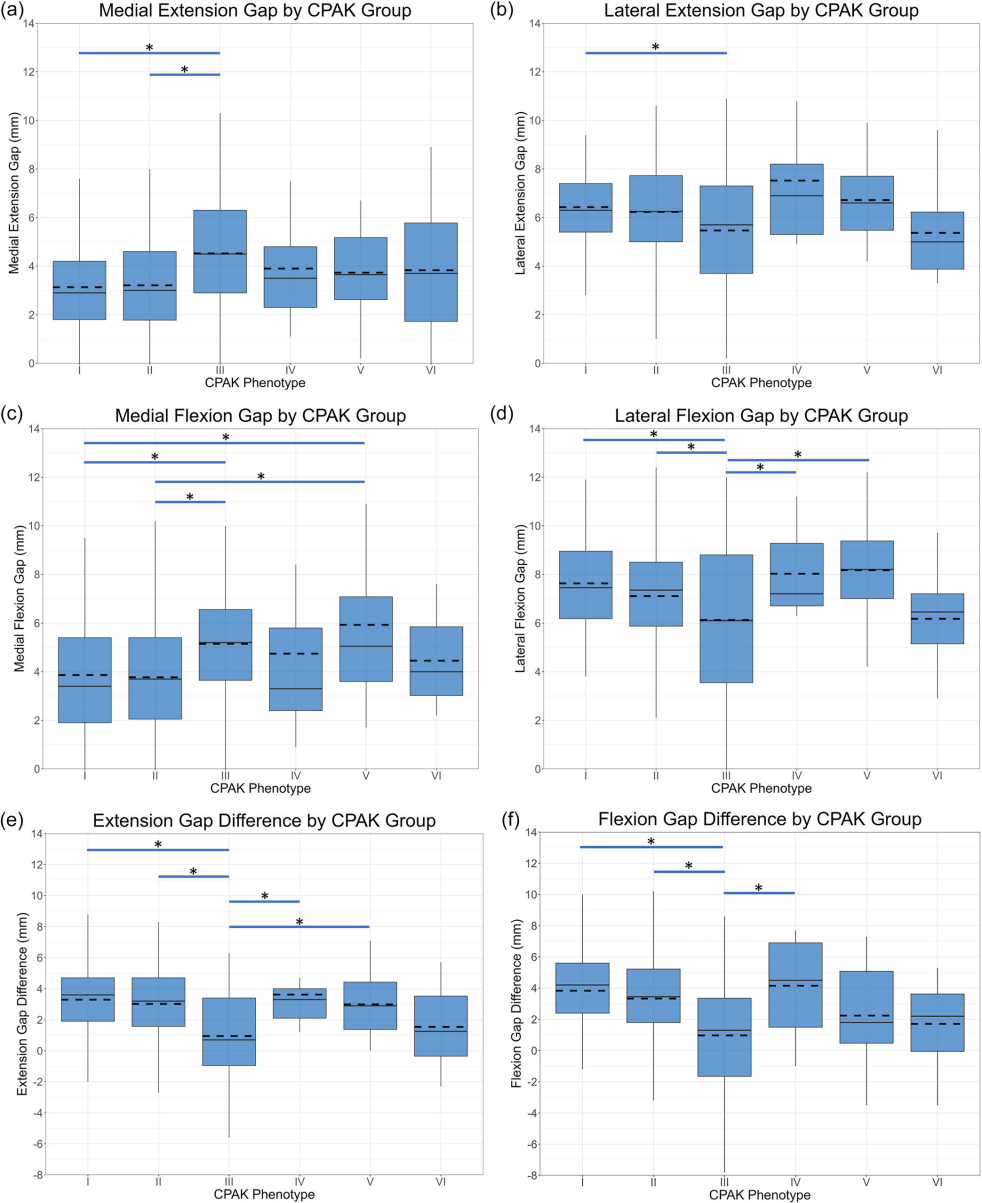
Distributions of the joint gap measurements by CPAK type. For each box, the horizontal solid line represents the median, while the horizontal dotted line indicates the mean. The lower edge of the box corresponds to the 25th percentile (Q1), and the upper edge corresponds to the 75th percentile (Q3), illustrating the interquartile range (IQR) (Q3–Q1). The whiskers (vertical solid lines) extend from the lower and upper boundaries of the box to the smallest and largest values within 1.5 times the IQR from these boundaries, respectively. *Indicates statistically significant (*p* < 0.05) differences between the two groups at either end of the blue line. (a) Medial extension gap measurements across CPAK phenotypes, with significant differences between phenotypes I and III, and II and III. (b) Lateral extension gap measurements across CPAK phenotypes, with significant differences between phenotypes I and III. (c) Medial flexion gap measurements across CPAK phenotypes, with significant differences between phenotypes I and III, I and IV, II and III, and II and V. (d) Lateral flexion gap measurements across CPAK phenotypes, with significant differences between phenotypes I and III, II and III, III and IV, and III and V. (e) Extension gap difference measurements across CPAK phenotypes, with significant differences between phenotypes I and III, II and III, III and IV and III and V. (f) Flexion gap difference measurements across CPAK phenotypes, with significant differences between phenotypes I and III, II and III, and III and IV. CPAK, coronal plane alignment of the knee.

The mean values and distributions of the intercompartmental and composite joint gaps in both extension and flexion across the different CPAK groups are presented in Table 3 and Figure 2. A general trend was observed in which medial gaps increased, and lateral gaps decreased from CPAK types I to III and IV to VI, with some statistically significant differences observed. Notably, CPAK types I and III differed significantly in all four intercompartmental gap measurements (medial and lateral, in both extension and flexion) as well as in extension and flexion gap differences. CPAK types II and III also exhibited statistically significant differences in medial extension and flexion gaps, lateral flexion gap, and both extension and flexion gap differences. While additional differences were noted involving types IV and V, the small sample sizes in CPAK groups IV, V, and VI limited the strength of statistical inferences for these phenotypes.

**Table 3:** Mean and standard deviation of the axial and sagittal measurements of the full study cohort and each CPAK group from I to VI.

Measurement	All groups (*n* = 433)	CPAK group					
		I (*n* = 109)	II (*n* = 180)	III (*n* = 95)	IV (*n* = 9)	V (*n* = 20)	VI (*n* = 16)
Medial extension gap (mm)	3.5 ± 2.1	3.1 ± 2.1	3.2 ± 2.0	4.5 ± 2.1	3.9 ± 2.0	3.7 ± 1.8	3.8 ± 2.7
Lateral extension gap (mm)	6.1 ± 2.2	6.4 ± 2.1	6.2 ± 2.1	5.5 ± 2.4	7.5 ± 2.8	6.7 ± 1.6	5.4 ± 1.8
Medial flexion gap (mm)	4.2 ± 2.5	3.9 ± 2.7	3.8 ± 2.1	5.2 ± 2.4	4.7 ± 3.6	5.9 ± 3.1	4.5 ± 1.8
Lateral flexion gap (mm)	7.1 ± 2.6	7.7 ± 2.3	7.1 ± 2.4	6.1 ± 3.1	8.9 ± 3.1	8.2 ± 2.4	6.2 ± 1.8
Average extension gap (mm)	4.8 ± 1.7	4.8 ± 1.6	4.7 ± 1.7	5.0 ± 1.7	5.7 ± 2.2	5.2 ± 1.4	4.6 ± 2.0
Average flexion gap (mm)	5.7 ± 2.0	5.8 ± 2.0	5.4 ± 1.8	5.6 ± 2.2	6.8 ± 3.0	7.1 ± 2.4	5.3 ± 1.3
Extension gap difference (mm)	2.6 ± 2.7	3.3 ± 2.6	3.0 ± 2.4	1.0 ± 2.8	3.6 ± 2.1	3.0 ± 2.0	1.5 ± 2.3
Flexion gap difference (mm)	2.9 ± 3.3	3.8 ± 3.0	3.3 ± 2.8	1.0 ± 3.5	4.2 ± 3.1	2.2 ± 2.9	1.7 ± 2.5

Abbreviation: CPAK, coronal plane alignment of the knee.

### Univariate regression analysis

Statistically significant linear relationships were observed between all four intercompartmental joint gap measurements and aHKA, but these relationships were all weak (< 0.4 in magnitude). The correlations suggest a weak trend towards medial extension and flexion gaps increasing, and lateral extension and flexion gaps decreasing as aHKA increases. Only the medial flexion gap displayed a statistically significant correlation with JLO (*r* = 0.15, *p* = 0.002).

Of the composite gap measurements, the extension gap difference and flexion gap difference were weakly correlated to aHKA (*r* = −0.31, *p* < 0.001 and *r* = −0.32, *p* < 0.001). This suggests that as aHKA increases, the medial joint gap increases relative to the lateral compartment, to a similar effect in extension and flexion. The average flexion gap measurement also displayed a weak yet statistically significant relationship with JLO (*r* = 0.15, *p* = 0.003).

The Pearson's correlation coefficients between all joint gap measurements and aHKA and JLO are summarised in Table 4.

**Table 4:** Pearson correlation coefficients of the joint gap measurements to both the arithmetic hip‐knee‐ankle angle (aHKA) and the joint line obliquity (JLO).

Variable	Correlation coefficient (*r*)	
	aHKA	JLO
Medial extension gap	0.22**	0.07
Lateral extension gap	−0.15*	0.01
Medial flexion gap	0.21**	0.15*
Lateral flexion gap	−0.21**	0.06
Average extension gap	0.03	0.06
Average flexion gap	−0.03	0.15*
Extension gap difference	−0.31**	−0.03
Flexion gap difference	−0.32**	−0.08

* Indicates *p* < 0.05.

** Indicates *p* < 0.001.

### Multivariate regression analysis

The multivariate linear regression revealed that all four intercompartmental joint gaps significantly influenced aHKA (Table 5). However, the model demonstrated limited explanatory power, with a residual standard error of 3.8° and an adjusted *R* ^2^ of 10.2%. For JLO, the multivariate regression results were weaker, explaining only 1.4% of the variance, with a residual standard error of 3.4° (Table 6). Notably, the medial flexion gap was the only statistically significant predictor of JLO.

**Table 5:** Results for the multivariate linear regression model evaluating the predictive relationship between the medial and lateral joint gaps in extension and flexion and arithmetic hip‐knee‐ankle angle (aHKA).

Variable	Coefficient	Standard error	*t* value	*p* value
(Intercept)	0.82	0.63	1.31	0.19
Medial extension gap	0.30	0.11	2.81	0.005
Lateral extension gap	−0.22	0.11	−2.12	0.03
Medial flexion gap	0.24	0.09	2.65	0.008
Lateral flexion gap	−0.25	0.09	−2.90	0.004

*Note*: The model explains 11.1% of the variance in aHKA (adjusted *R* ^2^ = 10.2%), with a residual standard error of 3.8° and *F* statistic of 13.3 (*p* < 0.001). With statistical significance at *p* < 0.05, all four intercompartmental joint gap measurements were significant predictors.

**Table 6:** Results for the multivariate linear regression model evaluating the predictive relationship between the medial and lateral joint gaps in extension and flexion and joint line obliquity (JLO).

Variable	Coefficient	Standard error	*t* value	*p* value
(Intercept)	171.84	0.57	300.25	<0.001
Medial extension gap	−0.08	0.10	−0.81	0.42
Lateral extension gap	−0.02	0.10	−0.22	0.82
Medial flexion gap	0.18	0.08	2.18	0.03
Lateral flexion gap	0.13	0.08	1.60	0.11

*Note*: The model explains just 2.3% of variance in JLO (adjusted *R* ^2^ = 1.4%), with a residual standard error of 3.4° and *F* statistic of 2.56 (*p* = 0.04). With statistical significance at *p* < 0.05, the medial flexion gap was the only significant predictor of JLO.

## DISCUSSION

This study found that CPAK phenotypes, and aHKA and JLO exhibited low predictability for medial, lateral and composite joint gaps in both extension and flexion. Although some statistically significant differences in joint gap measurements were observed between CPAK groups, the phenotypes did not demonstrate distinct joint gap profiles in extension and 90° flexion. Notably, significant differences in multiple joint gap measurements were found between CPAK types I and III and II and III, suggesting that these may be driven by differences in aHKA. While statistically significant linear correlations were observed between aHKA and medial gaps, lateral gaps, and gap differences, they were weak, with the highest Pearson's correlation coefficient magnitude of 0.32. Fewer statistically significant correlations were noted between JLO and joint gaps, with the medial and average flexion gaps displaying the largest coefficients (*r* = 0.15 for both). Multivariate regression analyses further underscored the limited relationship between joint gaps and aHKA and JLO, with joint gaps explaining a very small proportion of the variance in aHKA (10.2%) and JLO (1.4%). Overall, these findings suggest that CPAK groupings, aHKA and JLO provide limited insight into soft tissue behaviour and cannot reliably predict patient‐specific tibiofemoral joint gaps in extension and 90° flexion. Clinically therefore, following the CPAK principles will not always result in a balanced TKA. Surgeons still need to consider patient specific soft tissue characteristics as well as their balance target preferences in their approach to alignment.

While statistically significant differences were observed between CPAK types I and III and II and III for many of the joint gap measurements, the magnitude of these differences was generally small—often less than 2 mm—raising questions about their clinical relevance. Although there is currently no established minimum clinically important difference (MCID) for preoperatively measured joint gaps in TKA, intraoperative studies offer a useful reference point. Wakelin et al. demonstrated that patients with medial and lateral gaps, or mediolateral gap differences, within defined intraoperative balance windows experienced significantly better KOOS pain scores and were more likely to achieve the Patient Acceptable Symptom State (PASS) [32]. Importantly, their data shows that a change of more than 2 mm in gap size would shift over 50% of patients who initially met these balance targets outside of the optimal range. Based on this reference threshold, the small differences in mean intercompartmental and composite gap measurements observed between CPAK phenotypes in the current study are unlikely to be clinically meaningful. Grosso et al. observed that MPTA significantly influenced medial, but not lateral, extension and flexion distractibility in varus knees, while LDFA showed no statistically significant impact [10]. Since the MPTA and LDFA are used to determine the aHKA and JLO, which in turn define the CPAK groups, this may explain why the phenotypes did not display distinct joint gap profiles. However, Holland et al. analysed 1112 robotic TKAs and identified unique joint gap curve morphologies for CPAK groups I to VI from 0° to 110° flexion [13]. While they reported mean joint gaps at 10° intervals, no statistical testing validated the differences between groups. Unlike this study's preoperative stress radiographs on conscious patients, Holland et al. measured gaps intraoperatively after medial release and anterior and posterior cruciate ligament removal [13], factors which can impact the intercompartmental extension and flexion gaps [23, 29]. Therefore, further research is required to establish whether the joint gap morphologies for CPAK groups are truly distinct. Until this can be established, alternative approaches are required to preoperatively profile and plan for joint gaps during TKA.

While studies investigating the relationship between intercompartmental and composite joint gaps with aHKA and JLO are limited, our findings reflect weak but variable relationships between coronal alignment and joint gap behaviour. In varus knees, Graichen et al. observed that greater varus deformity was associated with statistically significant decreases in medial extension and flexion gaps and an increase in lateral extension gap, resulting in a reduced medial‐to‐lateral gap difference [8]. The extension gap difference was more strongly correlated to HKA (*r* = 0.79) compared to flexion (*r* = 0.40). In valgus knees, Eller et al. observed an increase in medial gaps and decrease in lateral gaps with increased valgus deformity [5]. They also observed stronger correlations between the extension gap difference and HKA (*r* = 0.82) relative to flexion (*r* = 0.21). These trends align with our study, where weak correlations were observed between aHKA and medial, lateral, and composite gaps, with coefficients ranging from −0.32 to 0.22, reflecting the limited predictive value of coronal alignment parameters. Furthermore, Grosso et al. observed increased medial distractibility in both extension and flexion as varus deformity increased [10]. While this contrasts the directionality of the results observed in the current study and by Graichen et al. [8], their result may be influenced by their definition of distractibility, which was the difference between the post‐tibial resection, distracted joint gap less the resected bone thickness. Nonetheless, all these studies emphasise the high inter‐patient variability in intercompartmental and composite joint gap measurements in extension and flexion with standard deviations of greater than 2 mm for each [5, 8, 10].

The weak results between coronal alignment parameters and joint gaps during both univariate and multivariate regression analyses suggest that other factors influence joint gaps apart from bone morphology and degree of deformity. One potential explanation is the dynamic behaviour of knee deformity during flexion, with as few as 14% of patients maintaining their coronal deformity throughout flexion range of motion [4]. Patient factors may also influence joint gap behaviour. In a cohort of neutral and varus knees, Graichen et al. observed that women had larger mean extension and flexion gaps than men (by 0.5 mm), while body mass index and age had minimal impact [8]. However, these variables did not influence gaps in valgus knees [5]. While further research is required to understand the variables that influence native joint gaps, we can conclude that joint gap behaviour is complex and cannot be effectively predicted by coronal alignment parameters such as aHKA and JLO.

Although the CPAK system offers a simplified method of classifying coronal knee alignment, its ability to predict soft tissue balance appears limited. This is likely due to its reliance on bone‐referencing measurements such as aHKA and JLO. Alternative systems, such as the Functional Knee Phenotypes proposed by Hirschmann et al., incorporate the HKA [12], which is directly influenced by joint spacing and has shown stronger associations with joint gaps [5, 8]. While further research is required, classification systems that consider HKA may offer improved predictive value for preoperative joint gaps and soft tissue behaviour.

### Limitations

This study has some potential limitations. While CPAK types VII, VIII and IX were excluded from most of the statistical analyses, they accounted for just 0.9% (4) patients. This reflects published statistical analyses [3, 13, 16] and proportions of diverse global populations [3, 19, 21, 22, 24, 26, 27, 28]. Additionally, the CPAK distribution in this study varied from the arthritic population assessed by MacDessi et al. [19]. This is likely due to the different imaging modalities used to assess bone morphology. The current study performs measurements using 3D anatomical analysis from CT scans, whereas MacDessi et al. obtained measurements from 2D antero‐posterior radiographs [19]. We believe that 3D anatomical analysis is the gold standard for TKAs and the CPAK distribution in the current study aligns with reported 3D analyses [3, 27]. The adjustments in joint gaps from the osteophyte correction algorithm are a best estimate and require further validation. Due to the imaging protocol, the joint gaps were measured at 0° and 90° of flexion. While computer navigation and robotic systems can measure gaps intraoperatively, they do not aid in preoperative planning. The patient cohort was predominantly Caucasian, and the results cannot be extrapolated to other ethnic groups. Clinical data regarding tibiofemoral instability were not uniformly available. As a result, joints with instability may have been included. This is a limitation of the analysis, as such cases may exhibit altered joint gap behaviour, potentially affecting the generalisability of the findings. Additionally, we are not able to present clinical outcomes for the cases nor PROMs. We are also not advocating or able to define joint gap targets but rather highlighting the population variability in soft tissue behaviour and its independence from distal femoral and proximal tibial anatomy. Surgeons need to be cognisant of the fact that blindly following bony morphology alone will not result in the same TKA gaps in all patients. Thus, restoring a patient's CPAK category, aHKA and JLO may not deliver the tibiofemoral balance that the surgeon has aimed for. Our findings further emphasise the need for methods to objectively measure soft tissue gaps and laxity and correlate these with patient outcomes. This will permit soft tissue parameters and alignment targets to be defined for individual patients, with the aim of improving TKA function and longevity.

## CONCLUSION

This study demonstrated that while the CPAK classification system and phenotypes are useful for describing bone‐referenced coronal knee anatomy, they do not effectively predict preoperative medial, lateral or composite joint gaps in extension and flexion within a TKA patient cohort. To achieve optimal joint balance in TKA, a thorough understanding of the soft tissue profile is essential. Given the lack of a reliable predictor for preoperative tibiofemoral joint gaps, direct assessment of these gaps is currently necessary to inform surgical planning. Further research is needed to develop a preoperative planning workflow that incorporates joint gap measurements as well as bone anatomy, aiming to enhance both joint balance and patient outcomes.

## AUTHOR CONTRIBUTIONS

**Ishaan Jagota**: Conceptualisation; formal analysis; investigation; methodology; project administration; visualisation; writing—original draft. **Rami M. A. Al‐Dirini**: Supervision; writing—review and editing. **Mark Taylor**: Supervision; writing—review and editing. **Joshua Twiggs**: Conceptualisation; methodology; supervision; writing—review and editing. **Brad Miles**: Conceptualisation; resources; supervision; writing—review and editing. **David Liu**: Conceptualisation; methodology; data curation; supervision; writing—review and editing.

## CONFLICT OF INTEREST STATEMENT

At the time of writing this manuscript, IJ, JT and BM were all employees of 360 Med Care, Mathys Orthopaedics and Enovis ANZ. This research was supported by 360 Med Care, Mathys Orthopaedics and Enovis ANZ. BM has stock or stock options in Enovis and 360 Med Care, has/does receive royalties from Enovis ANZ and Corin, and has been a paid consultant for 360 Med Care, Corin and Johnson & Johnson. MT and RA have received research support from 360 Med Care. DL has received royalties from Zimmer Biomet, research support from Zimmer Biomet, Depuy and Allay Therapeutrics, is a paid speaker for Zimmer Biomet, Depuy, and LifeHealthCare, is a paid consultant for Zimmer Biomet and Depuy and has stock or stock options in Naviswiss, Formus Labs Ltd, and ArthroLase Pty Ltd. DL is an unpaid consultant for Formus Labs Ltd and ArthroLase Pty Ltd, is on the editorial board for Knee Surgery and Related Research and for Arthroplasty, and is also a board member for the Asia Pacific Arthroplasty Society and the Knee Arthroplasty Subcommittee, SICOT.

## ETHICS STATEMEMT

Bellberry Human Research Ethics Committee (study number 201203710).

## Data Availability

Data are not publicly available.
